# From Assistance to Autonomy: Acceptability of Progressive Artificial Intelligence Integration in Facial Reconstructive Surgery—Protocol for Within-Subjects Vignette Experiment Among Romanian Adults

**DOI:** 10.3390/healthcare14183030

**Published:** 2026-09-15

**Authors:** Valeria Dina, Andrei-Paul Tent, Andrei-Teodor Maghiar, Ruxandra Florina Bodog, Titus-Andrei Grecu, Octavian-Marius Crețu, Laura Maghiar, Florian Bodog, Georgiana Albina Căiță, Mircea Ioan Șandor, Raul Chioibas, Ioan Lascăr

**Affiliations:** 1 Doctoral School, Faculty of Medicine, “Carol Davila” University of Medicine and Pharmacy, 050474 Bucharest, Romania; valeria.gorea@drd.umfcd.ro (V.D.); ioan.lascar@gmail.com (I.L.); 2 Department of Oral and Maxillo-Facial Surgery, Faculty of Medicine and Pharmacy, University of Oradea, Universității Street No. 1, 410087 Oradea, Romania; tent_andrei@yahoo.com; 3 Department of Surgical Disciplines, Faculty of Medicine and Pharmacy, University of Oradea, 1st December Square No. 10, 410073 Oradea, Romania; bodog.ruxandraflorina@student.uoradea.ro (R.F.B.); fbodog@uoradea.ro (F.B.); drcaitageorgiana@gmail.com (G.A.C.); msandor@uoradea.ro (M.I.Ș.); 4 Department of Surgery, Pelican Hospital, Corneliu Coposu Street No. 2, 410450 Oradea, Romania; 5 Doctoral School of Biomedical Sciences, Faculty of Medicine and Pharmacy, University of Oradea, Universității Street No. 1, 410087 Oradea, Romania; 6 County Clinical Emergency Hospital of Oradea, 410087 Oradea, Romania; 7 Department of Plastic Surgery, The Christie NHS Foundation Trust, 550 Wilmslow Road, Manchester M20 4BX, UK; titus.grecu@nhs.net; 8 Department of Surgery I—Clinic of Surgical Semiotics and Thoracic Surgery, “Victor Babeș” University of Medicine and Pharmacy Timișoara, Eftimie Murgu Square No. 2, 300041 Timișoara, Romania; octavian.cretu@umft.ro; 9 Center for Hepato-Biliary and Pancreatic Surgery, “Victor Babeș” University of Medicine and Pharmacy Timișoara, Eftimie Murgu Square No. 2, 300041 Timișoara, Romania; 10 Department of Psycho-Neurosciences and Rehabilitation, Faculty of Medicine and Pharmacy, University of Oradea, Universității Street No. 1, 410087 Oradea, Romania; laura.maghiar@uoradea.ro; 11 Department of Dermatovenerology, Clinical County Emergency Hospital Bihor, Gheorghe Doja Street No. 65, 410169 Oradea, Romania; 12 Department of Surgery I, Faculty of Medicine, “Victor Babeș” University of Medicine and Pharmacy Timișoara, Eftimie Murgu Square No. 2, 300041 Timișoara, Romania; 13 CBS Medcom Hospital, Popa Șapcă Street No. 12, 300047 Timișoara, Romania

**Keywords:** artificial intelligence, surgical planning, facial reconstructive surgery, acceptability, trust, risk perception, responsibility attribution, vignette experiment, algorithm aversion, study protocol

## Abstract

Artificial intelligence (AI) is entering surgical planning along a continuum that runs from imaging assistance to near-autonomous plan generation, and facial reconstructive surgery—where outcomes bear directly on identity and social functioning—is among the most sensitive settings for this transition. Existing acceptability research has largely compared AI-supported care with conventional care in binary designs, and Central and Eastern European populations remain underrepresented. This protocol describes a cross-sectional, within-subjects vignette experiment among Romanian adults recruited online (target *N* = 300 valid responses; minimum analyzable *N* = 140, raised by a pre-registered rule if the pilot’s inter-scenario correlation falls below the planning assumption). Each participant will read four hypothetical scenarios describing progressively autonomous AI involvement in facial reconstructive surgical planning: imaging assistance, decision support, outcome simulation, and autonomous planning. Participants are randomly allocated to one of the four sequences of a Williams balanced Latin-square design, so that the autonomy level is orthogonal to serial position and to first-order carry-over. After each scenario, they will rate acceptability, trust, and perceived risk (three seven-point Likert items each), responsibility attribution across four loci (surgeon, hospital, AI developer, unavoidable medical uncertainty), perceived AI control and perceived technical sophistication (manipulation checks), and scenario difficulty. Primary analyses are mixed repeated-measures ANOVAs with presentation sequence as a between-subjects factor, orthogonal polynomial (linear, quadratic, cubic) trend contrasts, and Holm-corrected pairwise comparisons, complemented by a position-adjusted linear mixed model; secondary analyses address responsibility migration and concentration, moderation by AI familiarity, and global implementation preferences. A pilot study (*N* = 50, allocated 1:1 to the ascending and descending sequences) will establish reliability, factor structure, comprehension, manipulation validity, and a first bound on order effects before pre-registration on the Open Science Framework and the start of data collection.

## 1. Introduction

### 1.1. Background

Artificial intelligence (AI) applications have expanded rapidly across plastic and reconstructive surgery, with systematic reviews documenting uses that span diagnosis, preoperative planning, intraoperative support, and outcome prediction [1,2,3]. Facial plastic and reconstructive surgery accounts for many of the most mature examples [4,5]. Deep learning systems now segment craniofacial structures on computed tomography (CT) with expert-level accuracy [6], localize three-dimensional cephalometric landmarks automatically [7], and quantify bone properties such as mineral density opportunistically from routine CT [8]. Beyond image analysis, AI models generate candidate surgical plans from cephalometric data [9], automate donor-site segmentation for virtual planning of facial reconstruction [10,11], and simulate three-dimensional postoperative facial appearance before an operation is performed [12,13]. At the far end of the continuum, autonomous robotic systems have completed soft-tissue surgical tasks under minimal human control in preclinical settings [14], and frameworks now grade surgical AI and robotic systems by their levels of autonomy, from passive assistance to conditional autonomy [15,16], with staged evaluation pathways proposed for their clinical introduction [17].

Facial reconstructive surgery is a particularly consequential setting for this transition. Because the face is central to personal identity, social interaction, and psychological well-being, decisions about facial reconstruction carry stakes that extend beyond function, and ethical analyses of AI in facial surgery emphasize consent, privacy, and the special status of facial data [18]. Whether progressively autonomous planning systems are adopted will depend not only on technical performance but also on their acceptability to prospective patients: attitudes, trust, perceived risk, and beliefs about who bears responsibility when errors occur shape patients’ willingness to consent, clinicians’ willingness to rely on algorithmic outputs, and the regulatory conditions under which such systems operate [19,20].

### 1.2. Public Attitudes Toward Medical AI

A substantial body of the recent literature has characterized public and patient attitudes toward clinical AI. Syntheses converge on a consistent pattern: the public is broadly receptive to AI as a support tool but insists on human involvement and personalized care [21,22]. Large surveys find that most patients expect AI to improve care while reporting substantial concerns about misdiagnosis, privacy, and the erosion of the patient–clinician relationship [23], that comfort is conditional on physician supervision of the final decision [24,25], and that trust is lowest precisely for surgical applications [26]. Experimental work shows that acceptance of AI-based care rises when physicians endorse and explain the system [27,28], and that patients react negatively when AI use is not disclosed or when AI and clinician recommendations conflict [29,30]. A recent multinational hospital survey spanning 43 countries confirmed predominantly favorable views combined with a marked preference for explainable, physician-supervised AI [31]. Within surgery specifically, patients presenting to a surgical clinic reported low familiarity with and limited trust in AI involvement in their care [32], and users evaluating AI-enabled decision aids valued them for shared decision-making rather than autonomous decision-making [33]. Vignette-based population experiments identify system reliability and transparency as the leading drivers of support [34], and AI familiarity, education, and health literacy recur as determinants of acceptance [35,36].

### 1.3. Trust, Algorithm Aversion, and Responsibility

Two research streams supply the study’s theoretical structure. The first concerns algorithm aversion and appreciation: systematic reviews and integrative syntheses show that lay evaluations of algorithmic advice depend on the task, the decision stakes, the visibility of algorithmic error, and the degree of human control retained, with aversion most pronounced in consequential, subjective, or embodied domains [37,38]. In medicine, reluctance to rely on algorithmic care has been traced in part to an illusory sense of understanding of human decision-making relative to algorithmic decision-making, and it can be attenuated by explanation [28]; explainability, however, is not sufficient on its own, since explanation quality can either build or erode trust [39]. Trust research in healthcare AI—for both the public and clinicians—identifies perceived reliability, transparency, workflow fit, and accountability as recurring determinants [19,40].

The second stream concerns responsibility for AI-involved errors. Surveys show the public predominantly holds physicians responsible for harms arising in AI-assisted care, while physicians more often assign liability to developers and institutions [41]; experimental work with mock jurors indicates that clinicians who follow standard-of-care AI advice are not judged more harshly [42], and that institutions attract greater blame after AI-involved adverse events unless clinicians visibly engage with the system’s outputs [43]. Conceptual analyses describe how causal, moral, and legal responsibility diffuse across clinicians, developers, and institutions in AI-supported care—the “problem of many hands”—and warn of emerging responsibility gaps [44,45]. Normative work argues that the clinician’s role in the loop should amount to substantive co-reasoning rather than nominal veto power [46,47], a position echoed by the human-oversight obligations that the EU Artificial Intelligence Act imposes on high-risk systems [20,48]. The evolving European liability landscape—including gaps identified in the proposed AI liability directives and the reconfiguration of the doctor–patient relationship under the AI Act—makes the allocation of responsibility for surgical AI a live regulatory question [49,50,51]. However, empirically, how laypeople re-allocate responsibility as AI autonomy increases has scarcely been quantified, particularly with within-person designs; a recent vignette study among physicians found that they retained personal moral responsibility while endorsing institutional co-responsibility [52], but comparable public data along a graded autonomy continuum are lacking.

### 1.4. Adjacent Consumer-Facing Health AI: Capability Advances and the Acceptability Lag

A third body of work, published largely in engineering rather than health-services venues, sets the technical horizon against which public attitudes must be read, and shows why acceptability evidence lags capability. Hu et al. [53] use global and marginal contrastive clustering to recognize faces in partially aligned, multi-view consumer-device data, achieving robust identification where views are incomplete or unpaired. This is the same class of representation that makes automated craniofacial analysis feasible outside a controlled radiology setting, an indication of how readily facial data captured on ordinary consumer devices can be organized and matched at scale. Yang et al. [54] describe a consumer-centric digital-twin framework for coronary artery disease in which a continuously updated patient model performs risk stratification and generates individualized management proposals, with explainability layered over the predictions. Structurally, this is our Scenario D—a machine-generated plan that a clinician reviews rather than composes—transplanted to cardiology; plan generation is therefore being engineered for consumer-facing deployment across specialties, not confined to surgical research settings. Yang et al. [55] address the layer beneath both, using reinforcement learning with adversarial data augmentation to detect distributed denial-of-service attacks against connected consumer healthcare devices; the availability and integrity of that layer are preconditions for any clinical claim built on top of it, and they are also, in the patient’s view, part of what is being trusted.

Read together, these studies establish that the capability frontier—biometric facial representation, autonomous plan generation, and the connected infrastructure that carries both—is advancing fastest on the consumer side, where devices and data leave the clinical setting, and the person concerned has the least oversight. What none of them establish, and what remains unresolved, is whether the people whose faces, physiology, and devices are involved regard these arrangements as acceptable, whom they hold responsible when such systems err, and at which point along the autonomy continuum their acceptance breaks down. Technical validation is reported as accuracy against benchmarks and as robustness under attack; acceptability is a distinct empirical quantity with its own measurement requirements, and it cannot be inferred from either. Two consequences follow for the present protocol. First, the autonomy levels examined here are not speculative: each corresponds to a capability already realized or under active development, so the attitudes measured are attitudes toward systems that patients may encounter. Second, the design isolates the quantity the technical literature leaves unmeasured—it holds the clinical task constant, varies only the degree of machine autonomy, and records acceptability, trust, perceived risk, and responsibility attribution within person, so that the location and shape of any decline in acceptance can be estimated rather than assumed.

### 1.5. Knowledge Gaps and Rationale

Three gaps motivate the present protocol. First, most acceptability studies compare AI-supported care with conventional care in binary designs [21,22]; such designs cannot reveal where along the continuum from assistance to autonomy acceptance begins to erode, whether the decline is linear or threshold-like, or how trust, risk perception, and responsibility attribution reconfigure across intermediate levels of autonomy of the kind now documented in facial surgical planning [9,13,15]. Second, the evidence base is geographically skewed toward North America, Western Europe, and East Asia [21,31]. Central and Eastern Europe is sparsely represented: the available regional studies concern physicians’ perceptions in Romania [56,57], Romanian medical students [36,58], and general or patient populations in Poland [59,60]; none examines graded surgical AI autonomy in a general adult population. Attitudes in this region cannot simply be extrapolated from findings elsewhere, given differences in health-system experience, institutional trust, and digital-health exposure [59,60]. Third, and cutting across both, the acceptability evidence has not kept pace with the capability frontier reviewed in Section 1.4: the forms of AI involvement whose acceptability is least well characterized—machine-generated plans reviewed rather than composed by a clinician, built on facial data captured and carried by consumer devices—are precisely those now being engineered for deployment [53,54,55], so the interval between technical feasibility and evidence on public acceptance is widening rather than closing.

### 1.6. Objectives and Hypotheses

The primary objective is to evaluate the attitudes of Romanian adults recruited online—acceptability, trust, risk perception, and responsibility attribution—toward four progressive scenarios of AI implementation in facial reconstructive surgery. The secondary objectives are (i) to analyze the moderating effect of AI familiarity on acceptability across autonomy levels; (ii) to investigate how responsibility attribution (surgeon, hospital, AI developer, unavoidable medical uncertainty) varies across scenarios; (iii) to explore the moral–legal distinction in responsibility attribution; (iv) to identify the preferred model of AI implementation in facial reconstructive surgery; and (v) to quantify the influence of presentation sequence on all primary outcomes and to estimate the autonomy effect adjusted for serial position.

Four hypotheses will be tested. H1. Acceptability and trust decrease significantly as the level of AI autonomy in the surgical decision increases. H2. Perceived risk increases as human control over the decision process decreases. H3. Responsibility attribution shifts progressively from the surgeon toward the AI developer as algorithmic autonomy increases. H4. Prior familiarity with AI moderates the relationship between implementation scenario and acceptability, such that participants familiar with AI show a smaller decline in acceptability for high-autonomy scenarios; H4 is treated as partially exploratory, given the power constraints described in Section 2.5. H1–H3 fix the expected direction but not the functional form: each is tested by an omnibus effect of autonomy level followed by the full set of orthogonal polynomial contrasts, so that threshold-like or otherwise non-monotonic patterns are detected rather than forced into a linear trajectory (Section 2.9.1). A design-validation hypothesis is tested alongside them, namely that responses do not depend on the sequence in which the scenarios were presented; it is evaluated as the scenario × sequence interaction, and a non-null result is treated as evidence of order dependence to be reported and adjusted for, not as a substantive finding about autonomy.

## 2. Materials and Methods

### 2.1. Study Design

This is a cross-sectional, vignette-based experiment with a four-level within-subjects (repeated-measures) design. Vignette experiments combine the internal validity of experimental manipulation with survey feasibility and are an established method for isolating drivers of health-related judgments [61]; their design and analysis follow current methodological guidance for vignette data [62]. Each participant reads and evaluates all four scenarios of AI implementation (A–D), presented in a counterbalanced sequence. The within-subjects structure makes each respondent their own control, eliminating inter-individual variability from between-scenario comparisons and substantially increasing statistical power relative to a between-subjects design of the same size [63]. The presentation sequence is counterbalanced. The survey platform allocates participants at random, in equal proportions and independently of any participant characteristic, to one of the four sequences of a Williams (balanced Latin-square) design: A → B → C → D, B → D → A → C, C → A → D → B, and D → C → B → A. In this square, every scenario occupies every serial position exactly once, and every ordered pair of scenarios occurs exactly once, so that autonomy level is orthogonal both to serial position and to first-order carry-over, and an additive model containing a four-level autonomy effect and a four-level serial-position effect is fully identified. The theoretically motivated ascending sequence and its exact mirror image are retained as two of the four arms. A fixed ascending presentation would have mirrored the progressive continuum under investigation and given every participant an identical evaluation context, but it would have left order, carry-over, and fatigue effects inseparable from the autonomy gradient; because H1–H3 concern precisely that gradient, internal validity was given priority over uniformity of context. Counterbalancing also makes the embedded manipulation check diagnostic rather than merely descriptive: perceived AI control should track the described autonomy level within every sequence, whereas an anchoring or fatigue artifact would instead track serial position—a distinction that a monotonic manipulation-check profile obtained under a fixed order cannot draw. Presentation sequence is therefore retained as a between-subjects factor in all primary models, a second manipulation check (perceived technical sophistication) separates autonomy from technical complexity, and a per-scenario difficulty item indexes fatigue (Section 2.6, Section 2.7, Section 2.8, and Section 2.9.1). The study involves no medical, pharmacological, or surgical intervention. Reporting of the completed study will follow current recommendations for effect-size-based reporting [64] and, for the web-survey component, the CHERRIES checklist as operationalized in recent methodological guidance [65]. Publication of the protocol in advance of data collection is intended to reduce publication bias and analytic flexibility [66,67].

### 2.2. Experimental Vignettes

The four vignettes describe distinct configurations of AI involvement in facial reconstructive surgical planning, varying both the AI’s task (imaging analysis, decision support, outcome simulation, autonomous planning) and the surgeon’s level of supervision (Table 1). Each configuration corresponds to a capability documented in the current literature: anatomical structure analysis from CT [6,8], algorithmic generation of candidate surgical plans [9], three-dimensional simulation of postoperative facial appearance [12,13], and highly autonomous planning of the kind anticipated by surgical-autonomy frameworks [14,15]. All vignettes open with an identical premise (the respondent needs facial reconstructive surgery following an injury) and are written in parallel second-person wording of comparable length and reading difficulty, so that scenario content—not framing—drives differences in responses. Because the four configurations differ in what the AI does as well as in how much the surgeon decides, a perceived difference between them could in principle reflect technical sophistication rather than autonomy. Three features are intended to keep the two apart. First, every vignette closes by stating explicitly how the final surgical plan is reached and by whom. In Scenarios A–C, a single clause of parallel construction names the surgeon as the decision-maker (making all decisions, choosing among the proposed options, or deciding the final plan after reviewing the simulations); in Scenario D, the closing sentences state that the surgeon reviews the AI’s plan but rarely modifies it. What varies is therefore the decisional locus, not the computational task. Each comprehension check is built on that same feature: the decision-maker in Scenarios A and B; the role of the system in Scenario C (whose decisional clause is functionally equivalent to Scenario B’s, so that the role is what distinguishes the two); and the origin of the plan actually followed in Scenario D. Second, the vignettes are matched on the quantity of technical detail supplied—one sentence describing the system’s input and output in each case—so that described sophistication does not rise in step with autonomy. Scenario C specifies a generative three-dimensional simulation, computationally the most elaborate output in the set, yet leaves the decision entirely with the surgeon; Scenario D describes a comparatively conventional optimization whose distinguishing feature is decisional rather than technical. Third, two manipulation checks follow every scenario: MAN1, perceived control of the AI over the final decision, and MAN2, perceived technical sophistication of the system. The manipulation is treated as valid only if MAN1 orders the scenarios as intended; MAN2 verifies that sophistication does not itself increase monotonically with autonomy and enters a pre-specified sensitivity analysis as a covariate, so that the autonomy effect can be estimated with perceived sophistication held constant (Section 2.9.1). The full vignette texts are provided in Appendix A.

### 2.3. Participants: Eligibility Criteria

Inclusion criteria: age ≥ 18 years; current residence in Romania; ability to read and complete an online questionnaire in Romanian; and informed consent expressed by ticking the consent checkbox on the first page of the instrument. Exclusion criteria applied at screening: age < 18 years, residence outside Romania, or refusal of consent. Exclusion criteria were applied at data cleaning, following current recommendations for careless-responding control [68,69]: failure of both embedded attention checks; incorrect response to the comprehension check—after re-presentation of the scenario (second attempt)—for at least two of the four scenarios; invariant response patterns (the same response selected for every Likert-type item in the questionnaire); and duplicate records (identical response patterns across all variables, submitted consecutively).

### 2.4. Recruitment and Setting

Participants will be recruited online through social networks, forums, and professional groups in Romania, and through the mailing lists of higher-education institutions, with the approval of the respective administrations. No financial compensation is offered; participation is entirely voluntary, and participants are informed that they can stop at any time. The resulting sample is a non-random convenience sample. The study makes no claim of national representativeness: the demographic structure of the sample (including residence environment and development region) will be reported against reference data from the Romanian National Institute of Statistics, and generalizability will be discussed explicitly as a limitation. The validity of between-scenario comparisons—the study’s primary objective—is protected by the within-subjects design, in which every participant evaluates all four scenarios, so that comparisons across autonomy levels do not depend on sample composition or recruitment method. Feasibility is supported by comparable online surveys of attitudes toward AI in healthcare, which recruited samples of approximately 200–2700 respondents from online populations [24,27,34].

### 2.5. Sample Size and Power

Sample size is justified a priori following current recommendations [70], with all calculations derived analytically from noncentral *F*, t, and chi-square distributions at a two-tailed significance threshold of α = 0.05 and a target power of 1 − β = 0.80, reproducible in G*Power 3.1 (“ANOVA: Repeated measures, within factors”) and consistent with recent tutorials on power analysis for repeated-measures designs [63]. The primary test is a repeated-measures ANOVA (four within-subject levels) on each composite score. Assuming a correlation between repeated measurements of ρ = 0.50 (a typical value for repeated attitudinal ratings, to be verified in the pilot), the minimum sample is 24 participants for medium effects (*f* = 0.25) and 138 for small effects (*f* = 0.10); under the conservative assumption ρ = 0.30, these become 32 and 193, respectively. The minimum analyzable threshold is therefore set at *N* = 140 valid participants, sufficient to detect even small between-scenario effects under realistic conditions. Because these thresholds depend on ρ, an adaptive rule is pre-registered for the case in which the pilot estimate falls outside the assumed ρ = 0.30–0.50 range. The pilot returns $\hat{\rho }$ with a 95% confidence interval, and every threshold is recomputed at its lower bound, ρ_L_, which is the conservative choice. The number of valid participants required to detect *f* = 0.10 at power 0.80 is 274, 247, 220, 193, 165, and 138 for ρ_L_ = 0, 0.10, 0.20, 0.30, 0.40, and 0.50, respectively; an intermediate ρ_L_ is rounded down to the nearest tabulated value, so that the rule is fully determined in advance for any estimate the pilot can return. Two consequences follow. The recruitment target does not change because the requirement is a decreasing function of ρ and reaches only 274 in the theoretical worst case of ρ_L_ = 0; *N* = 300 preserves power ≥0.80 against small between-scenario effects for every value ρ can take, and the target is in any case set by the person-level analyses described below, which do not depend on ρ. The analytic floor does change: it is redefined as the value tabulated above for the observed ρ_L_, so that it remains at the pre-specified *N* = 140—rounded up from 138—only when ρ_L_ ≥ 0.50 and rises to 165, 193, 220, 247, or 274 as ρ_L_ falls to 0.40, 0.30, 0.20, 0.10, or 0. A ρ_L_ below 0.10 would in addition indicate that the composites are unstable across repetitions rather than that participants are inconsistent, and would therefore also trigger re-inspection of pilot reliability and of the measurement-invariance assumptions before the main study is launched. Any application of this rule, and the $\hat{\rho }$ that triggered it, will be recorded in the pre-registration and reported in the results article.

**Figure 1:**
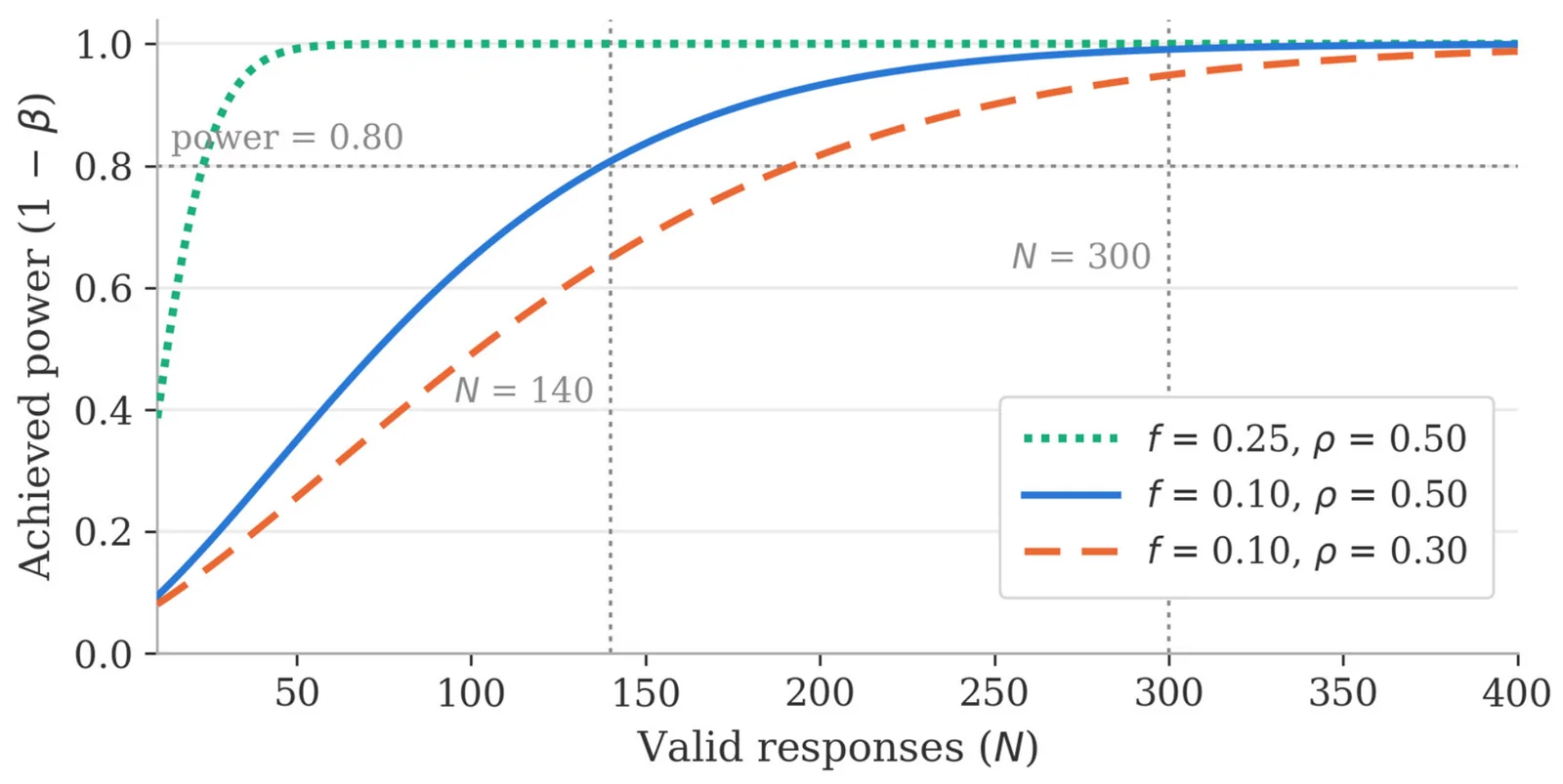
Achieved power of the primary repeated-measures ANOVA (four within-subject levels, α = 0.05) as a function of the number of valid responses, for small (*f* = 0.10, at ρ = 0.50 and ρ = 0.30) and medium (*f* = 0.25) effects. Dotted reference lines mark power = 0.80 and the two pre-specified thresholds (*N* = 140, minimum analyzable; *N* = 300, recruitment target). Curves are computed analytically from the noncentral *F* distribution. Entering presentation sequence as a between-subjects factor consumes three degrees of freedom from the error term and leaves these curves unchanged to within rounding.

The recruitment target is *N* = 300 valid participants. This target is dictated not by the within-subjects effects (for which power at *N* = 300 is 0.99 for small effects at ρ = 0.50, with a minimum detectable effect of *f* = 0.07), but by the person-level analyses: the familiarity moderation (minimum detectable interaction *f*^2^ = 0.037 at *N* = 300), the global preference test (minimum detectable *w* = 0.21 on 5 degrees of freedom), and descriptive analyses of demographic subgroups. A small interaction effect (*f*^2^ = 0.02) would require *N* ≈ 550; H4 therefore remains partially exploratory, and a non-significant result will not be interpreted as evidence of absence. At *N* = 300, dependent pairwise comparisons detect differences from *dz* = 0.16 (uncorrected) and *dz* = 0.20 (at the Bonferroni bound for six pairwise comparisons), so all six scenario contrasts can be treated as confirmatory. Counterbalancing entails no loss of sensitivity for the primary comparisons: with sequence entered as a four-level between-subjects factor, the autonomy main effect retains 3 degrees of freedom and the same noncentrality, so the minimum detectable effect at *N* = 300 remains *f* = 0.07. The scenario × sequence interaction—the direct test of order dependence—carries 9 degrees of freedom and a minimum detectable effect of *f* = 0.08 at ρ = 0.50 (*f* = 0.10 at ρ = 0.30), giving power of 0.95 against a small order effect of *f* = 0.10; the serial-position main effect in the additive model carries 3 degrees of freedom and matches the sensitivity of the autonomy effect itself. Order artifacts large enough to masquerade as the hypothesized autonomy gradient are therefore detectable, rather than being assumed away. In the pilot, by contrast, the ascending and descending arms (*n* = 25 each) bound order effects only at *f* = 0.17, which excludes medium-to-large artifacts but not small ones; the definitive test is the main-study interaction. Table 2 summarizes the power analysis, and Figure 1 shows achieved power as a function of the number of valid responses.

After expected attrition is taken into account—approximately 85% of link visitors consenting, 96% of starters eligible after screening, 75% of eligible respondents completing (drop-out conservatively inflated to 25% given the 15–20 min repeated-block format), and 90% of submissions surviving quality-based exclusions [68]—approximately 545 link accesses (≈334 submitted questionnaires) are required to yield 300 valid responses (Table 3). The stopping rule is accrual-based: data collection continues until a minimum of 300 valid responses is reached; the absolute analytic floor is *N* = 140—or the higher value returned by the adaptive rule above, should the pilot estimate of ρ require it—below which the main study is not analyzed, and collection is extended. Drop-out between the first and last scenario blocks will be monitored by serial position across all four sequences as a fatigue indicator.

### 2.6. Instrument and Measures

The instrument is a structured online questionnaire originally developed for this study, administered in Romanian in a single version distributed through a single link; the complete instrument (English translation) is reproduced in Appendix A. It comprises twelve sections: (1) informed consent and eligibility screening; (2) background and prior experience—AI familiarity (B1), experience with AI tools (B2), confidence in understanding medical information (B3), prior surgery and prior face/head/neck surgery (B4, B4a), healthcare contact frequency (B5), and trust in the national healthcare system (B6); (3) general attitudes toward AI in medicine (G1–G4, with G2 and G4 reverse-keyed for any composite); (4) the first attention check; (5–8) the four scenario blocks, administered in the sequence to which the participant was randomly allocated, each consisting of a vignette, a comprehension check, and an identical response block; (9) overall preferences and acceptability (P1–P4b); (10) the second attention check; (11) demographics (D1–D7: gender, age, education, occupational field, subjective financial situation, residence environment, development region); and (12) closing debriefing.

The per-scenario response block contains nine Likert items measuring the three primary dependent variables—acceptability (ACC1–ACC3), trust (TRU1–TRU3), and perceived risk (RSK1–RSK3)—followed by six responsibility items (RES1–RES4: surgeon, hospital, AI developer, unavoidable medical uncertainty; RES5: the surgeon’s moral duty to verify AI outputs; RES6: outcome-independent accountability), two manipulation-check items (MAN1: perceived AI control over the final surgical decision; MAN2: perceived technical sophistication of the system), and a perceived difficulty item (DIF1). All scaled items use 7-point end-anchored Likert response formats. Composite scores are computed as the arithmetic mean of their items after reverse-scoring RSK1 (8 − score), which is the only reverse-keyed item in the response block; higher risk scores indicate higher perceived risk. Treating item means on end-anchored 7-point scales as quasi-continuous interval variables is standard practice in experimental vignette research; ordinal-model sensitivity analyses are nevertheless planned in line with current guidance on Likert-based outcomes [62,71]. Table 4 summarizes the measurement structure; per-scenario variables carry the scenario suffix (e.g., ACC1_A, ACC1_B, ACC1_C, ACC1_D).

Because everyday usage collapses several distinct notions into one, the instrument separates them explicitly, and the header shown to participants states the intended sense. RES1–RES3 measure agent-directed responsibility—how much of the responsibility for an error each party should bear—and appear, together with RES4, under a common header that states that responsibility may be shared and that each party is to be rated separately rather than as a share of a fixed total, so that both diffusion and concentration are expressible; the header also glosses responsibility as answering for the error and putting it right, expressly distinguishing it from being found at fault in court. RES4 is not an agent and captures the alternative attribution that an error reflects irreducible clinical uncertainty; it is analyzed separately and is excluded from the concentration index. RES5 isolates moral responsibility from legal fault by construction (“even if the surgeon were not legally at fault…”), and RES6 isolates accountability in its outcome-independent sense—whether answerability depends on how the operation turned out—which is precisely what separates accountability from outcome-based blame. Legal liability proper is not measured per scenario. It is asked once, globally, and separately from the ethical claim, as two items presented on the same page and scale: P4a, that the surgeon should always remain legally responsible for any surgical outcome, and P4b, that the surgeon should always remain ethically responsible for it. Keeping them apart matters because a single double-barreled item would force the two notions to move together and would conceal exactly the pattern the responsibility literature predicts—a surgeon held morally answerable for an outcome to which no legal fault attaches. Both are analyzed descriptively and against RES5 and RES6, and their difference within participants—the extent to which a respondent holds the surgeon morally but not legally answerable—is itself reported. They are not treated as within-scenario outcomes, because lay judgments of legal liability depend on doctrinal assumptions that a short vignette cannot supply and that vary with the respondent’s exposure to the legal system; treating such judgments as a repeated-measures outcome would attribute a precision to them that they do not have. The Romanian wording of these items avoids the terms “culpă” and “răspundere juridică” except where legal fault is explicitly intended, and the pilot cognitive interviews (Section 2.8) probe specifically whether participants read RES1–RES3 as moral answerability, as causal contribution, or as legal liability. If the interviews reveal systematic conflation, the header wording will be revised, and the items retested before pre-registration, and the change documented.

### 2.7. Data Quality Procedures

Four mechanisms protect data quality, consistent with current best-practice recommendations for careless-responding prevention and detection [68,69] and with multi-layered approaches to online-survey integrity [72]. First, two attention checks are embedded in the questionnaire: ATT1, an instructed-response item placed after the general attitudes section (participants are asked to select a specific scale point regardless of their opinion), and ATT2, a content check after the scenario blocks asking which type of surgery the scenarios described. Exclusion applies only when both checks are failed. Second, each vignette is followed by a single multiple-choice comprehension check targeting the scenario’s defining feature (who made the final decision, or what the AI’s role was). A first incorrect response triggers branching that re-presents the full scenario, after which the check is repeated; a second incorrect response flags that scenario as failed (CC_*x*_FAIL = 1), and participants failing at least two of the four scenarios are excluded. Third, invariant response patterns (identical responses to all Likert items, including across scenarios) and duplicate records are screened during data cleaning. Fourth, all closed questions are mandatory on the survey platform, structurally preventing item-level missingness in the dependent variables; “prefer not to answer” options exist only for selected background and demographic items (B4, B4a, B5, B6, D1, D6, D7) and are coded as a distinct category descriptively and as missing in inferential models; participants who decline the separate explicit consent for the two health-related items (Section 2.10) are not presented with items B4 and B4a, and those records are coded in the same way. The platform does not record completion time, so data quality relies on the checks above rather than on a temporal filter; this is declared as a limitation. Finally, each participant’s sequence allocation (SEQ) is made at the point of entry to the scenario blocks and written to the submitted record, and the platform maintains its page counters separately for each sequence. Drop-out can therefore be attributed to a sequence and a serial position even where no submission is stored. Balance across the four sequences is monitored during accrual and reported in the participant-flow diagram, and section-wise drop-out is tabulated by serial position as well as by scenario, so that fatigue can be distinguished from a reaction to a particular scenario. The comprehension checks remain valid under counterbalancing because each is bound to the scenario it immediately follows, and ATT2 concerns content common to all four scenarios.

### 2.8. Pilot Study and Instrument Validation

**Figure 2:**
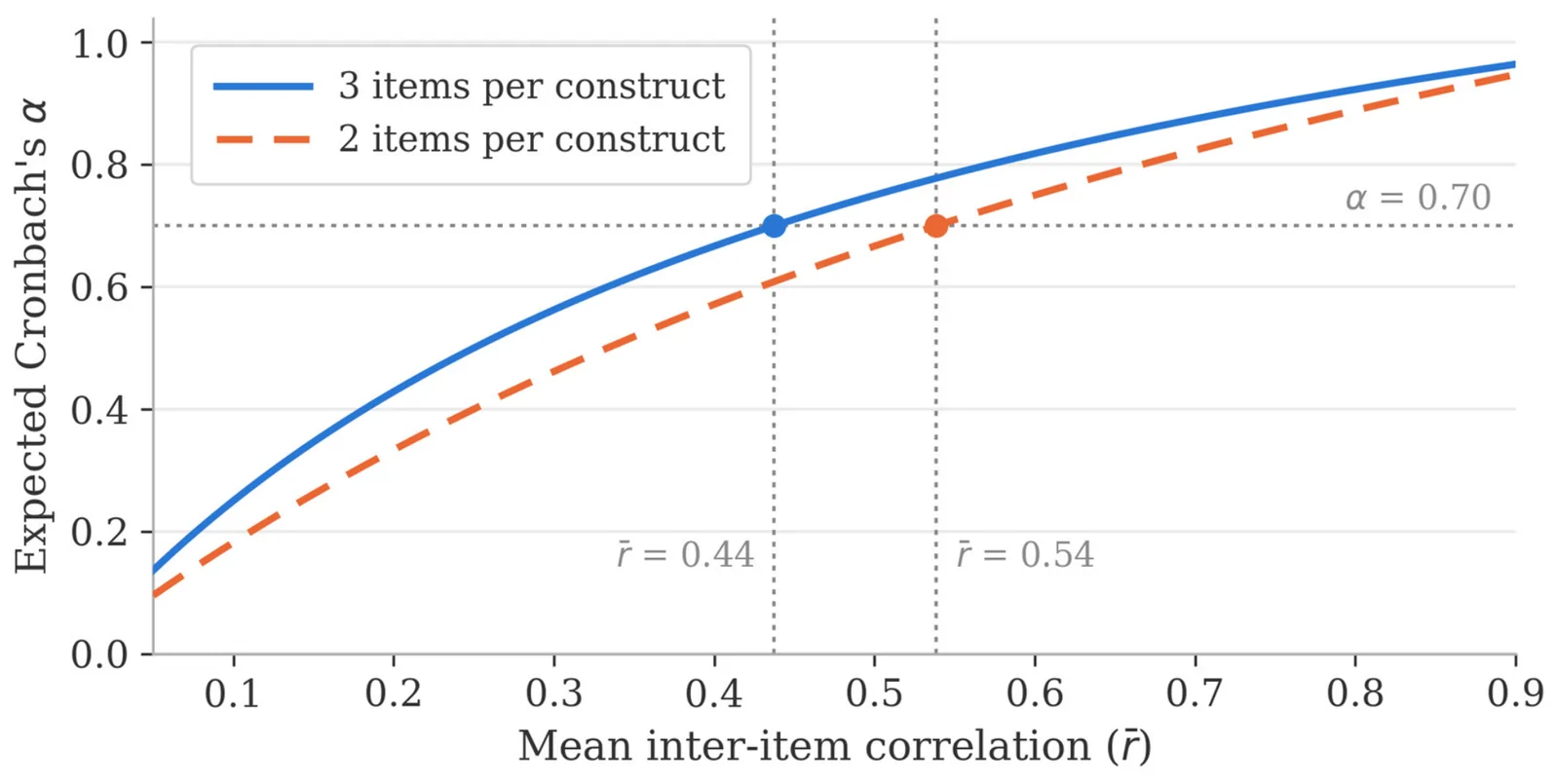
Expected internal consistency (Cronbach’s α) as a function of the mean inter-item correlation, for two- versus three-item composites (generalized Spearman–Brown relation). With three items per construct, the pre-specified threshold α = 0.70 is reached at $\bar{r}$ = 0.44 (markers), versus $\bar{r}$ = 0.54 with two items—the structural advantage of the instrument’s three-item composites.

The instrument will be validated in a pilot study (*N* = 50, not included in the main sample, allocated 1:1 to the ascending sequence A → B → C → D and to its exact reverse D → C → B → A) before the main study is launched. The pilot will estimate (i) internal consistency—Cronbach’s α and McDonald’s ω with 95% confidence intervals for each three-item composite, computed separately per scenario, against a threshold of ≥0.70, with ω reported alongside α in line with current psychometric guidance [73]; with three items per construct, α ≥ 0.70 requires a mean inter-item correlation of only $\bar{r}$ ≥ 0.44 under the generalized Spearman–Brown relation (Figure 2), a realistic requirement for parallel items; (ii) inter-item correlations (flagged at r > 0.90 for redundancy or r < 0.20 for heterogeneity) and corrected item-total correlations (threshold ≥ 0.40), plus the correlation between repeated measurements, reported with a 95% confidence interval, to verify the ρ ≈ 0.50 planning assumption and, where it is not met, to trigger the adaptive sample-size rule of Section 2.5; (iii) the factor structure of the nine response items, via exploratory factor analysis (principal-axis extraction, oblique rotation, sampling adequacy KMO ≥ 0.70, Bartlett’s test of sphericity, parallel analysis as the retention criterion)—an anticipated risk is that acceptability and trust items form a single factor, in which case the two constructs will be merged into a single “trust–acceptance” score and the analysis plan adjusted transparently before pre-registration, with this decision taken exclusively on pilot data; (iv) first-attempt comprehension-check pass rates per scenario (threshold ≥ 70%); (v) manipulation validity—MAN1 scores must order the scenarios from A to D in both pilot arms, while MAN2 (perceived technical sophistication) must not increase monotonically with autonomy; if it does, the vignettes will be reworded to equalize the described sophistication of the systems and the check repeated before pre-registration; (vi) fatigue—perceived difficulty (DIF1) and section-wise drop-out across serial positions in both arms; (vii) cognitive interviews with 5–8 participants to identify confusing wording, probing in particular whether RES1–RES6 are read in the intended senses of moral answerability, outcome-independent accountability, and legal fault (Section 2.6); and (viii) a first bound on order effects—the two pilot arms are compared on the three composites and on MAN1 by a scenario × order mixed ANOVA (*n* = 25 per arm; minimum detectable interaction *f* = 0.17 at ρ = 0.50), which can exclude medium-to-large order artifacts but not small ones, the definitive test being the main-study interaction. The four-sequence randomizer used in the main study is implemented in the same pilot build. Because the pilot itself exercises only two of the four arms, the randomizer is verified there by simulated allocation, which also confirms that the allocated sequence and the serial position of each scenario are stored correctly. A ninth objective is included: (ix) the keying and dimensionality of the four general-attitude items (G1–G4), since G3 is a caution item that the present scoring treats as positively keyed while G2 and G4 are reversed; the scoring rule for that block is fixed on pilot data before pre-registration, and if the four items do not form a single interpretable dimension they will enter the person-level analyses individually rather than as a composite. Because a sample of 50 is at the lower limit for stable factor solutions, the pilot EFA is treated as provisional: the factor structure will be re-examined by confirmatory factor analysis on the main sample (*N* = 300), with acceptable fit defined a priori as CFI/TLI ≥ 0.95, RMSEA ≤ 0.06, and SRMR ≤ 0.08. Because the same nine items are answered four times, longitudinal measurement invariance across the four scenario repetitions (configural, metric, and scalar) will also be tested on the main sample; at least partial scalar invariance is required for the between-scenario comparison of composite means, and any non-invariant item will be reported and handled by partial-invariance modeling. Pilot results will be reported in the methods section of the results article.

### 2.9. Statistical Analysis

#### 2.9.1. Primary Analyses

Each composite score (acceptability, trust, perceived risk) will be analyzed by a mixed repeated-measures ANOVA, with autonomy level (four scenarios) as the within-subjects factor and presentation sequence (four Williams sequences) as the between-subjects factor. The Greenhouse–Geisser correction will be applied when Mauchly’s test indicates a sphericity violation (Huynh–Feldt for ε between 0.75 and 1), and the multivariate approach (Pillai’s criterion) will be reported in parallel as a sphericity-robust check. H1 and H2 will be tested with within-subjects trend contrasts across the autonomy gradient. Because acceptance need not fall in a straight line, the complete set of orthogonal polynomial contrasts—linear, quadratic, and cubic—is pre-specified, and each is reported together with the proportion of the omnibus effect it accounts for. A purely linear profile leaves the quadratic and cubic components negligible, whereas a step between two adjacent levels appears as a large linear component accompanied by a substantial quadratic or cubic one. Because every monotone step is linear-dominant, the polynomial decomposition establishes departure from a straight line but cannot by itself locate the break; the break is identified only from the Holm-corrected adjacent-pair contrasts (A–B, B–C, C–D). A quadratic-dominant profile with a small linear component would instead indicate a non-monotone pattern, which the hypotheses do not predict and which would be reported as such. A non-monotonic or threshold pattern will be reported as such rather than summarized by the linear trend alone. Post hoc comparisons use dependent-samples *t*-tests for the six scenario pairs with Holm’s sequential correction, applied within each hypothesis-defined family of tests in line with current guidance on multiplicity control [74]. Effect sizes will be reported as partial η^2^ for omnibus tests and Cohen’s dz for pairwise comparisons, with 95% confidence intervals, following effect-size-centered reporting recommendations [64]. Assumption checks include graphical inspection of difference-score distributions; as a distributional robustness analysis, the Friedman test with Holm-corrected Wilcoxon signed-rank pairwise comparisons will be reported alongside each primary test—supplemented by ordinal (cumulative-link) models as sensitivity analyses for the Likert-based outcomes [71]—and the concordance of parametric and nonparametric conclusions stated explicitly. Order dependence is tested directly as the scenario × sequence interaction; a non-significant interaction, together with equivalence of the four sequence-specific profiles, supports pooling across sequences. Independently of that test, the autonomy effect is re-estimated from a linear mixed model with random intercepts per participant and additive fixed effects of autonomy level and serial position (1–4). The Williams square renders this model identified, so the estimate is adjusted for arbitrary—not merely linear—serial-position and fatigue effects, while first-order carry-over is balanced by the design rather than modeled; position-adjusted and unadjusted estimates are reported side by side, and any material divergence between them is treated as evidence of an order artifact and interpreted accordingly. Perceived difficulty (DIF1) and perceived technical sophistication (MAN2) are each added as time-varying covariates in further sensitivity models, addressing fatigue and the possibility that responses track the apparent complexity of the system rather than its autonomy. Finally, between-person heterogeneity in response profiles is examined by adding a random slope for the linear autonomy contrast and testing the improvement in fit by a likelihood-ratio test; a substantial random-slope variance indicates that an average trajectory conceals distinct response patterns and licenses the exploratory typology described in Section 2.9.2. Because a non-significant test is not evidence of absence, a smallest effect size of interest of *dz* = 0.20—the value detectable at the Bonferroni bound for the six pairwise contrasts at *N* = 300—is fixed in advance, and equivalence testing by two one-sided tests against that bound is reported alongside every null pairwise result, so that any claim that two adjacent scenarios do not differ rests on an equivalence test rather than on the absence of significance.

#### 2.9.2. Secondary and Exploratory Analyses

(i) Moderation (H4): the scenario × AI familiarity (B1, centered) interaction, via repeated-measures ANCOVA or a linear mixed model with random intercepts per participant, the recommended framework for vignette data [62]; presentation sequence is retained as a between-subjects factor here, as in every repeated-measures model in this protocol. (ii) Person-level multiple regression: mean acceptability across scenarios predicted by AI familiarity, general AI attitudes, and demographics (reporting *B*, *SE*, β, 95% CI, R^2^, VIF). (iii) Responsibility migration (H3): a 4 × 4 mixed ANOVA (scenario × actor, RES1–RES4, both within-subjects; presentation sequence between-subjects) with Greenhouse–Geisser correction; the scenario × actor interaction is the direct test of H3. Because H3 is confirmatory, it receives the same order controls as H1 and H2: the sequence terms are inspected first, and the migration estimate is repeated in a linear mixed model containing an additive serial-position term. (iv) Responsibility concentration: a normalized Herfindahl–Hirschman index computed per scenario from the relative weights of the three responsible actors (surgeon, hospital, AI developer; RES4—unavoidable medical uncertainty—is not a responsible actor and is analyzed separately), compared across scenarios by mixed repeated-measures ANOVA with sequence as a between-subjects factor; low values index diffusion of responsibility across actors, operationalizing the “problem of many hands” described in the responsibility literature [44,45]. (v) RES5 (moral duty) and RES6 (outcome-independent accountability): mixed repeated-measures ANOVAs across scenarios with sequence as a between-subjects factor, Holm-corrected within the family. (vi) Global preference (P1): a goodness-of-fit chi-square with standardized residuals over six analysis categories on 5 degrees of freedom. The six categories are the four scenarios, “I would prefer AI not to be involved in any way,” and a combined “no clear preference” category; the last pools the item’s two undecided options (“I have no preference” and “I cannot decide”), which are combined a priori because neither expresses a preference between scenarios and each is expected to be sparse. Consistency between stated preference and each participant’s maximum-acceptability scenario is reported descriptively. (vii) Exploratory: P4a and P4b (the surgeon’s permanent legal and, separately, moral responsibility) analyzed descriptively, compared with each other by a dependent-samples test on the within-person difference, and correlated with RES5/RES6; free-text concerns (P2) summarized by brief thematic analysis. (viii) Sensitivity analyses: primary analyses repeated (a) excluding participants with a single attention-check failure (who are retained under the primary rule) and (b) excluding participants with any comprehension failure, to demonstrate that conclusions do not depend on the cleaning criteria [69]. (ix) Order effects: descriptive profiles by sequence, the scenario × sequence interaction with its effect size and 95% confidence interval, and sequence-specific manipulation-check profiles. (x) Response-pattern typology (exploratory): where the random-slope test of Section 2.9.1 indicates heterogeneity, individual acceptability trajectories across autonomy levels are classified by latent-class growth analysis, with class enumeration by BIC and entropy and a minimum class size of 5% fixed in advance, and class membership described against AI familiarity, healthcare-system trust, and demographics; no inferential claim is attached to this analysis. (xi) Healthcare-system trust (B6) enters the person-level regression of (ii) as a covariate and is examined as an exploratory moderator of the autonomy effect, alongside healthcare contact frequency (B5) and prior surgical experience (B4, B4a). (xii) Subgroup analyses, all of which are secondary or exploratory, are governed by a minimum-cell rule fixed in advance: inferential tests within a subgroup are reported only where that subgroup contains at least 50 valid participants, the size at which a within-subject difference in *dz* = 0.40 is detectable at power 0.80; subgroups of 30–49 are reported descriptively with 95% confidence intervals and labeled as underpowered; subgroups below 30 are pooled into a residual category or not reported. A difference between subgroups is never inferred from a significant effect in one and a non-significant effect in another; only the formal interaction term, with its own confidence interval, supports such a claim. The set of moderators is fixed a priori, in the pre-registration, to seven: AI familiarity (B1), healthcare-system trust (B6), healthcare contact frequency (B5), prior surgical experience (B4/B4a), education (D3), age (D2), and field of activity (D4). Results are reported for all seven irrespective of significance, and no further moderator is added after the data are seen. (xiii) Missing data: because all closed items are mandatory, missingness in the dependent variables is structurally impossible within a submitted questionnaire. Two other forms of missingness remain. The first is unit-level, from questionnaires abandoned before submission. The platform is configured not to store partial submissions, so responses from abandoned questionnaires are neither retained nor analyzed (Section 2.10 and Appendix A.1). Attrition is therefore characterized from the platform’s page-level counters—how many participants reached each page—rather than from partial response data, and is reported as drop-out by serial position in the participant-flow diagram. Analyses are accordingly restricted to complete cases, that is, participants with all four scenario blocks, and no imputation is performed: with no partial records retained, there are no incomplete cases to impute. The second is item-level, on the background and demographic items that offer a “prefer not to answer” option or, in the case of B4 and B4a, are not displayed where the explicit health-data consent is declined; these are treated as a distinct category descriptively and as missing in inferential models, with a sensitivity analysis retaining the category in the person-level regression. Because complete-case analysis is not neutral when attrition is differential, drop-out is compared across the four presentation sequences, and any imbalance is reported and carried into the interpretation of the order analyses. All tests are two-tailed at α = 0.05, with exact *p*-values, effect sizes, and 95% confidence intervals reported for all tests, including non-significant ones. On closed 1–7 scales, no distribution-based outlier removal is applied.

Table 5 links each hypothesis or objective to its planned test and the coefficients that will be reported.

### 2.10. Ethical Considerations

The study will be conducted in accordance with the Declaration of Helsinki [75], the EU General Data Protection Regulation (GDPR, Regulation 2016/679), Romanian legislation on research involving human subjects, and the institution’s code of good research practice. The study is carried out within the principal investigator’s doctoral program at the “Carol Davila” University of Medicine and Pharmacy, Bucharest, and was approved by the University’s Research Ethics Committee (approval no. 17992, date 25 June 2026). Informed consent is obtained electronically: the first page of the instrument presents the full consent text (Appendix A.1), and continuation is possible only after ticking a consent checkbox confirming that the participant has read and understood the information, is at least 18 years old, and agrees to participate voluntarily. Because the study is fully online, collected without direct identifiers, and non-interventional, checkbox-based electronic consent without physical signature follows standard practice for survey research. The consent text also explains that, because no identifier is recorded, individual responses cannot be traced or withdrawn after submission, and that questionnaires abandoned before completion are not stored and are not used in the analysis. Minors are excluded by screening; institutionalized persons, detainees, and persons unable to consent independently are not enrolled. Consistent with evidence that patients value disclosure of AI involvement in care [29], a final debriefing page informs participants that the scenarios are hypothetical, that in current Romanian clinical practice final surgical decisions belong to the physician and highly autonomous AI systems of the kind described are not in use, and provides contact details for questions or post-publication results.

Two items in the background section of the instrument (Section 2.6) require separate treatment under data-protection law. B4 asks whether the participant has ever undergone a surgical intervention and, where the answer is affirmative, B4a asks whether that intervention involved the face, head, or neck. These are data concerning health within the meaning of Article 4(15) of the GDPR and therefore fall within the special categories of Article 9(1). The two items are retained because prior experience of surgery, and of facial surgery in particular, is a plausible determinant of the attitudes under study and is required for the interpretation of the responsibility measures. They are processed on the basis of the participant’s explicit consent under Article 9(2)(a). Accordingly, the consent page names these two items specifically, states that they concern health, states that answering them is optional, and requires a separate affirmative tick for the processing of health-related answers in addition to the general consent tick (Appendix A.1); participants who do not give this second consent take part in the study without being shown B4 and B4a. No other item falls within Article 9(1): the remaining background, attitudinal, and demographic items concern opinions, experience, education, occupational field, subjective financial situation, residence environment, and development region. No genetic or biometric data are collected, nor is any data revealing racial or ethnic origin, political opinions, religious or philosophical beliefs, trade-union membership, health beyond the two items named, sex life, or sexual orientation. Item D1 records self-described gender for sample description only, offers a “prefer not to answer” option, and is not used to infer any special category. The randomization of presentation sequence, the two additional items (B6 and MAN2), and the revised consent wording described above have been prepared as an amendment to the approved protocol for submission to the Research Ethics Committee, and neither the pilot nor the main data collection will begin before that amendment is approved.

### 2.11. Data Management

Data are collected exclusively through an online survey platform configured to collect no direct identifiers (no names, e-mail addresses, or account authentication). The instrument will be administered on [platform name and version to be inserted], hosted in [hosting location to be inserted]. The controller for the purposes of the GDPR is the “Carol Davila” University of Medicine and Pharmacy, Bucharest, within whose doctoral program the study is conducted; the platform operator acts as a processor under a data-processing agreement, and the health-related items processed under Art. 9(2)(a) are stored on the same terms as all other responses. At export, each record receives a row-identification code unrelated to participant identity, and the dataset includes the quality-indicator flags (ATT1_FAIL, ATT2_FAIL, CC_A_FAIL–CC_D_FAIL) needed for transparent application of the exclusion criteria, together with the allocated presentation sequence (SEQ) and the serial position of each scenario, which the order analyses require. Data are stored on the platform’s secured servers, protected by encryption and account-level access control; access is restricted to the principal investigator and the scientific coordinator. Anonymized data are exported in CSV and SPSS (.sav) format for analysis; analysis files contain no personal identifiers. Anonymized data will be retained for a minimum of 5 years after publication, and the raw platform data will be deleted upon completion of the analysis. The legal basis for processing is the participant’s consent (Art. 6(1)(a) GDPR). Two items—B4 and B4a, on previous surgery and on previous surgery involving the face, head, or neck—constitute data concerning health and therefore fall within the special categories of Art. 9(1) GDPR; they are processed on the basis of the explicit consent provided for in Art. 9(2)(a), obtained by a separate affirmative tick as described in Section 2.10, and are held within the same identifier-free record as every other response. The records are identifier-free rather than anonymous at the moment of collection—no direct identifier is recorded, but the responses of a single participant are held together as one submission on the platform—and the Art. 6(1)(a) and Art. 9(2)(a) bases are relied upon for that collection and storage phase. The exported analysis dataset carries only row-identification codes and no link back to the platform submissions, and once the raw platform data are deleted on completion of the analysis, no re-identifiable copy remains. No other special category of data within the meaning of Art. 9 GDPR is collected. Anonymized data, the instrument, and the analysis scripts will be shared openly on the Open Science Framework (OSF) so that every reported coefficient can be independently reproduced.

### 2.12. Pre-Registration and Study Status

The study will be pre-registered on OSF before data collection begins; pre-registration constrains analytic flexibility and selective reporting and calibrates the confidence that readers can place in confirmatory claims [67]. The pre-registration will include the analysis plan, exclusion criteria, hypotheses, and sample-size justification [70], and will distinguish confirmatory analyses (H1–H3) from the partially exploratory moderation analysis (H4) and the exploratory analyses (P2, P4a, P4b); any subsequent deviations from the plan will be declared and justified in the results article. The OSF registration DOI will be reported in the results article. At the time of submission of this protocol, ethics approval for the original protocol has been obtained, the amendment described in Section 2.10 has been prepared for submission, and data collection has not started; the pilot study and pre-registration precede the main data collection.

The pre-registration also specifies in advance how departures from the plan are handled. Any deviation—a change in model, of exclusion rule, or of composite structure, including the merger of the acceptability and trust composites should the pilot factor analysis require it—will be reported in a dedicated deviations table in the results article, giving the pre-registered decision, the change made, the reason for it, and the result of the analysis as originally planned alongside the result as revised, so that the influence of the deviation on the conclusions is visible rather than asserted to be absent. Every table and figure will label its analyses as confirmatory (H1–H3 together with the manipulation and order checks), partially exploratory (H4), or exploratory (the response-pattern typology, the subgroup analyses, P2, and P4a/P4b); no exploratory result will be described in the language of hypothesis testing, and no confirmatory label will be applied retrospectively to an analysis that was not pre-registered. The handling of missing data and of participant exclusions is pre-specified in Section 2.3, Section 2.7, and Section 2.9.2, and participant flow from link access to analyzed sample—including the number excluded under each criterion, the number analyzed in each presentation sequence, and the reasons for attrition—will be reported in a flow diagram. Pilot-based decisions (the factor structure, the ρ-driven sample-size rule of Section 2.5, and any rewording of the vignettes or of the responsibility headers) are taken exclusively on pilot data and are locked before the pre-registration is submitted; the pilot dataset and the pilot analysis code will be deposited with the pre-registration so that the sequence of decisions is auditable.

## 3. Expected Results

Based on the algorithm-aversion and trust studies [19,37,38], we expect acceptability and trust to decline—and perceived risk to rise—across the autonomy gradient from Scenario A to Scenario D, with the largest drop anticipated at the transition to autonomous planning (Scenario D), where the surgeon’s corrective role is described as residual. Whether that decline is a gradient or a step is an open question that the design is equipped to address: a step would show as a substantial quadratic or cubic component alongside the linear one, and its location would be identified by the adjacent-pair contrasts, which we expect to place the largest single drop at C–D. Nor do we assume the pattern is homogeneous across participants; a substantial random-slope variance would itself be informative, indicating that an average trajectory conceals distinct groups of respondents rather than describing a shared response. The expectation of decline is, in any case, consistent with survey evidence that public comfort is conditional on physician supervision [24,25,31]. For responsibility attribution, we expect the surgeon to be assigned the dominant share in low-autonomy scenarios [41], with a progressive migration toward the AI developer—and possibly a diffusion of responsibility across actors, indexed by falling Herfindahl concentration—as autonomy increases [44,45]. We expect the manipulation check (perceived AI control) to order the scenarios from A to D within each of the four presentation sequences, validating the experimental gradient independently of serial position, and we expect no scenario × sequence interaction. Should one nevertheless appear, the position-adjusted mixed-model estimate becomes the primary basis for interpreting H1–H3, and the magnitude of the order effect will itself be reported as a finding about the stability of vignette-based autonomy judgments. Given the design’s sensitivity (power of 0.99 against small within-subjects effects at ρ = 0.50, and 0.95 at ρ = 0.30, for the target sample), a null result for a primary hypothesis would place a tight upper bound on the size of any autonomy effect rather than leaving the question open; the pre-specified equivalence tests of Section 2.9.1 make that bound explicit rather than infer absence from non-significance [70]. The moderation hypothesis (H4) is powered only for medium-small interactions and will be interpreted with corresponding caution. Results will inform the ordering of public concerns along the assistance-to-autonomy continuum and identify which implementation configurations are publicly acceptable entry points for surgical AI in Romania.

## 4. Discussion

This study will provide, to our knowledge, the first experimental evidence on the acceptability of graded—rather than binary—AI autonomy in facial reconstructive surgery in a Central or Eastern European population. Its principal contributions are fourfold. First, the four-level autonomy gradient can localize where public acceptance erodes and establish whether the decline is linear or threshold-like—a question that binary AI-versus-clinician designs cannot answer [21,22] and that matters practically now that assistance, decision-support, simulation, and autonomous-planning capabilities all exist in facial surgical practice or preclinical development [9,13,14,15] and are being engineered for consumer-facing deployment in adjacent specialties [54], on infrastructure for which facial-data handling and security are themselves active engineering problems [53,55]. Second, the study offers a within-person account of responsibility migration across autonomy levels, including both a concentration index that captures the diffusion of responsibility across actors and items that separate moral from legal accountability; these data speak directly to the responsibility-gap debate [44,45,46] and to the human-oversight and liability questions raised by the EU regulatory framework [48,49,50]. Third, the study extends a geographically skewed evidence base [31] with general-population data from Romania, complementing the region’s existing clinician- and student-focused studies [36,56,57,58] and general-population work from Poland [59,60]. Fourth, the counterbalanced within-subjects structure allows the autonomy effect to be separated from serial position and from first-order carry-over, the principal internal-validity threat in graded-vignette designs and one that this literature has more often acknowledged than addressed; the study therefore yields not only an estimate of where acceptance erodes but an empirical bound on how much of any observed gradient is an artifact of the order in which the scenarios were read. The findings are relevant to surgeons and hospitals considering AI-supported planning, to developers designing the human-oversight configuration of their systems [17], and to regulators calibrating accountability frameworks for AI-involved surgical care [20,51].

The design has several strengths. The within-subjects structure yields high statistical power: small effects are detectable from *N* = 138–193 depending on assumptions, and power reaches 0.99 at the target *N* = 300 [63]. Three-item composites permit proper reliability estimation with both α and ω [73]. The manipulation check allows the autonomy gradient to be validated empirically, and counterbalancing to a Williams square makes that validation diagnostic, since a manipulation-check profile that holds within every sequence cannot be produced by a monotone order artifact. The scenario × sequence interaction supplies a direct and adequately powered test of order dependence (minimum detectable *f* = 0.08). Comprehension checks with scenario re-presentation, two attention checks, and invariant-pattern screening implement current recommendations for online data quality [68,69,72], and a mandatory pilot and OSF pre-registration protect against post hoc analytic flexibility [67]. Publishing the protocol itself contributes to transparency and reduces publication bias [66].

Several limitations are accepted by design and will be declared in the results article. First, the sample is a self-selected online convenience sample: results describe Romanian adults active in digital environments—likely younger and more educated than the general population—and do not support national generalization; the sample structure will be reported against National Institute of Statistics reference data. Second, the vignettes are hypothetical, and the distance between a vignette judgment and a decision taken in a consulting room is not merely one of degree. Participants evaluate a scenario in which they are not injured, not in pain, not facing a scheduled operation, and not choosing between real surgeons. They bear no consequences for the judgment they record, receive no counseling, and cannot ask questions. Above all, they are not exposed to the most influential element of real surgical decision-making: a conversation with the clinician who would operate, whose endorsement and explanation are known to shift acceptance of AI-supported care substantially [27,28]. Stated acceptability under these conditions is best read as a measure of prior disposition toward a described scenario rather than as a prediction of consent behavior. The ordering of the scenarios is likely to be more robust than the absolute levels, which could be lower than those a counseled patient would report or higher if hypothetical risk is discounted. This is a standard limitation of vignette methodology [61,62], and the remedies lie outside the present design: replication among patients actually scheduled for facial reconstructive surgery, and ultimately observation of consent decisions where such systems are in use. Third, presentation sequence is counterbalanced but not thereby rendered irrelevant. The Williams square balances serial position and first-order carry-over, so that the autonomy effect is estimable free of both, but no four-sequence design balances higher-order carry-over—a participant’s evaluation of the third scenario being shaped jointly by the first two—and contrast effects that depend on which scenario was seen immediately before therefore remain possible. The scenario × sequence interaction, the position-adjusted model, and the difficulty covariate bound the magnitude of what remains; they do not remove it, and a large residual would warrant a between-subjects replication in which each participant evaluates a single scenario. Fourth, demand and contrast effects are inherent to within-subjects designs: seeing all scenarios, participants may infer the study objective and exaggerate differentiation between scenarios; comparisons remain internally valid, but absolute difference magnitudes warrant caution. Fifth, all measures share a common method and a single measurement occasion, in a rapidly evolving attitude domain [38]. Sixth, the survey platform does not record completion time, so quality control relies on embedded checks rather than temporal filters [69]. Seventh, the demographic and familiarity subgroups formed within a convenience sample of 300 will differ substantially in size, and several will be small; the minimum-cell rule of Section 2.9.2, and the requirement that subgroup differences be inferred only from formal interaction terms, are intended to forestall the familiar error of asserting a difference from a significant effect in the larger subgroup together with a non-significant one in the smaller. None of these limitations is unusual for online vignette experiments, and declaring them in advance is intended to keep the eventual findings interpretable at their proper scope.

The Romanian setting is a substantive element of the design rather than merely a limit on generalizability. Attitudes toward algorithmic involvement in surgery are formed in relation to the health system a person actually uses: the frequency and character of their contact with it, the access difficulties they may have encountered, their confidence that an adverse outcome would be investigated and remedied, and the trust they place in physicians and hospitals as institutions rather than as individuals. Regional evidence points to a combination of marked interest in digital health and comparatively low institutional trust in Central and Eastern Europe [56,57,58,59,60], and that combination could push acceptability in either direction: low trust in human institutions may make an algorithmic second opinion attractive, while the same low trust may make the prospect of a machine decision that no one answers for more threatening. Rather than assume one reading, the instrument records trust in the healthcare system (B6), healthcare contact frequency (B5), prior surgical experience (B4, B4a), residence environment (D6), and development region (D7), and carries them into the person-level analyses, so that the mechanism can be examined within the sample. The findings will nonetheless describe Romanian adults active in digital environments. Comparison with the Polish general-population studies [59,60] and with the multinational hospital survey [31] will be made descriptively and with attention to differences in instrument and sampling, and extrapolation to other health systems—including those operating under the same EU liability framework but with different national malpractice regimes [49,50,51]—would require replication rather than assumption.

## 5. Conclusions

This protocol specifies, in advance of any data collection, the design, measurement structure, power justification, quality-control procedures, and complete analysis plan for a counterbalanced within-subjects vignette experiment on the acceptability of progressively autonomous AI in facial reconstructive surgery among Romanian adults. These decisions are fixed a priori and bound through pilot validation and OSF pre-registration, and the presentation sequence is counterbalanced so that the autonomy gradient is separable from the order in which the vignettes are read. The study is therefore designed to yield interpretable, reproducible evidence on three questions: where public acceptance of surgical AI erodes along the assistance-to-autonomy continuum; how responsibility for AI-involved errors is assigned across surgeons, institutions, and developers; and which implementation configurations the Romanian public regards as acceptable entry points for AI in facial reconstructive surgery.

## Figures and Tables

**Table 1:** The four experimental scenarios. The labels A–D denote autonomy level, not position in the sequence; scenarios are administered in one of four counterbalanced sequences (see Section 2.1).

Scenario	Label	Role of the AI System	Role of the Surgeon
A	Imaging assistance	Analyzes the CT scan and highlights anatomical structures (e.g., bone density, tissue layers)	Independently interprets the AI output and makes all decisions on the surgical plan
B	Decision support	Proposes three possible surgical plans, each with different advantages and disadvantages	Reviews all options, selects the most suitable plan, and may adjust it
C	Outcome simulation	Generates 3D simulations of postoperative facial appearance for each surgical option	Reviews the simulations, discusses them with the patient, and decides the final plan
D	Autonomous planning	Generates a complete, optimized surgical plan	Reviews the plan before surgery; modifications to the AI proposal are rare

**Table 2:** Minimum detectable effects (power = 0.80, α = 0.05).

Test	Parameters	Minimum Detectable Effect
RM-ANOVA, 4 within-subject levels	*N* = 24, ρ = 0.50	*f* = 0.25 (medium)
RM-ANOVA, 4 within-subject levels	*N* = 140, ρ = 0.50	*f* = 0.10 (small)
RM-ANOVA, 4 within-subject levels	*N* = 300, ρ = 0.50	*f* = 0.07
Dependent pairwise comparison	*N* = 300, α = 0.05	*dz* = 0.16
Dependent pairwise comparison	*N* = 300, α = 0.05/6	*dz* = 0.20
Scenario × familiarity interaction (*df* = 3)	*N* = 300, α = 0.05	*f*^2^ = 0.037
Goodness-of-fit chi-square, preference (*df* = 5)	*N* = 300, α = 0.05	*w* = 0.21
Autonomy main effect, sequence as between-subjects factor (*df* = 3)	*N* = 300, ρ = 0.50	*f* = 0.07
Scenario × sequence interaction—order test (*df* = 9)	*N* = 300, ρ = 0.50	*f* = 0.08
Scenario × sequence interaction—order test (*df* = 9)	*N* = 300, ρ = 0.30	*f* = 0.10
Pilot order bound, ascending vs. descending arm (*df* = 3)	*N* = 50, ρ = 0.50	*f* = 0.17
Dependent comparison within a demographic subgroup	*n* = 50, α = 0.05	*dz* = 0.40

Abbreviations: RM-ANOVA, repeated-measures analysis of variance; *N*, number of valid participants; *n*, number of participants per pilot arm or subgroup; ρ, correlation between repeated measurements; α, significance threshold; *df*, degrees of freedom; f and *f*^2^, Cohen’s effect-size indices for *F* tests; *dz*, Cohen’s effect size for dependent-samples comparisons; *w*, effect size for the goodness-of-fit chi-square test.

**Table 3:** Projected recruitment funnel.

Stage	Assumed Pass Rate	Estimated Number
Link accesses	—	≈545
Begin questionnaire (consent given)	85% of accesses	≈463
Eligible after screening (items S1, S2)	96% of starters	≈445
Completed (submitted) questionnaires	75% of eligible	≈334
Valid after quality exclusions (ATT, CC, invariant, duplicates)	90% of submitted	≈300

Abbreviations: S1 and S2, the two screening items of the instrument (Appendix A.1); ATT1 and ATT2, attention checks; CC_A–CC_D, comprehension checks.

**Table 4:** Measurement structure of the instrument.

Construct/Variable	Items	Scale	Role in Analysis
Acceptability	ACC1–ACC3 (mean), per scenario (×4)	Likert 1–7	Primary DV (H1), within-subjects
Trust	TRU1–TRU3 (mean), per scenario (×4)	Likert 1–7	Primary DV (H1), within-subjects
Perceived risk	RSK1 (reversed), RSK2, RSK3 (mean), per scenario (×4)	Likert 1–7	Primary DV (H2), within-subjects
Responsibility attribution	RES1–RES4, per scenario (×4)	Likert 1–7	Secondary DV (H3), within-subjects
Responsibility principles	RES5, RES6, per scenario (×4)	Likert 1–7	Secondary DV
Global preference	P1	7 response options (6 analysis categories)	Secondary DV
General acceptability	P3	Likert 1–7	Secondary DV
AI familiarity	B1	Likert 1–7	Moderator (H4)/covariate
General AI attitudes	G1–G4 (G2, G4 reversed)	Likert 1–7	Covariates
Experience and demographics	B2–B5, D1–D7	Mixed	Covariates/sample description
Manipulation check (perceived AI control)	MAN1, per scenario (×4)	Likert 1–7	Design validation
Scenario difficulty	DIF1, per scenario (×4)	Likert 1–7	Control covariate (fatigue)
Data quality	ATT1, ATT2, CC_A–CC_D	Categorical	Exclusion criteria
Trust in the healthcare system	B6	Likert 1–7	Covariate/exploratory moderator
Manipulation check (technical sophistication)	MAN2, per scenario (×4)	Likert 1–7	Design validation/covariate
Presentation sequence	SEQ	4 categories	Between-subjects design factor
Serial position of scenario	POS	1–4	Covariate (order, fatigue)

Abbreviations: DV, dependent variable; ACC, acceptability items; TRU, trust items; RSK, perceived-risk items; RES, responsibility items; MAN1 and MAN2, manipulation-check items; DIF1, perceived-difficulty item; ATT1 and ATT2, attention checks; CC_A–CC_D, comprehension checks; B1–B6, background items; G1–G4, general-attitude items; P1–P4b, preference items; D1–D7, demographic items; SEQ, presentation sequence; POS, serial position of the scenario; H1–H4, hypotheses 1–4.

**Table 5:** Hypothesis-by-hypothesis analysis plan.

Hypothesis/Objective	Planned Test	Reported Coefficients
Manipulation validation	Mixed RM-ANOVA on MAN1 (autonomy within × sequence between) + polynomial trend contrasts; the intended ordering A → D expected within every sequence	*F*, corrected *df*, *p*, partial η^2^; ε(GG)
H1. acceptability and trust decline with autonomy	Mixed RM-ANOVA per composite, sequence as between-subjects factor (Greenhouse–Geisser) + orthogonal polynomial trend contrasts (linear, quadratic, cubic); post hoc dependent *t*-tests, Holm correction	*F*, corrected *df*, *p*, partial η^2^ (90% CI); pairwise dz (95% CI)
H2. perceived risk increases with autonomy	Mixed RM-ANOVA (autonomy within × sequence between) + orthogonal polynomial trend contrasts; post hoc dependent *t*-tests, Holm correction	*F*, corrected *df*, *p*, partial η^2^; pairwise *dz*
H3. responsibility migrates surgeon → AI developer	4 × 4 mixed ANOVA (scenario × actor within, sequence between), Greenhouse–Geisser; the scenario × actor interaction is the test of H3; repeated with a serial-position term	*F*, corrected *df*, *p*, partial η^2^; ε(GG)
Responsibility concentration	Normalized Herfindahl–Hirschman index (RES1–RES3) per scenario, compared by mixed RM-ANOVA (sequence between)	Mean HHI per scenario; *F*, *p*, partial η^2^
Moral–legal distinction	Mixed RM-ANOVAs on RES5 and RES6 across scenarios (sequence between); Holm correction within family	*F*, corrected *df*, adjusted *p*, partial η^2^
H4. moderation by AI familiarity (partially exploratory)	RM-ANCOVA or linear mixed model with random intercepts: scenario (within) × B1 (centered), sequence between	F (or likelihood-ratio test), *p*, *f*^2^; simple slopes with 95% CI
Global preference (P1)	Goodness-of-fit chi-square over 6 analysis categories (2 undecided options pooled); standardized residuals; consistency with maximum-acceptability scenario, descriptive	χ^2^, *df*, *p*, *w*; standardized residuals
Predictors of acceptability	Person-level multiple regression: mean acceptability on familiarity, general attitudes, demographics	*B*, *SE*, β, 95% CI, R^2^, VIF
P2, P4a/P4b (exploratory)	Brief thematic analysis (P2); descriptive statistics, within-person P4a–P4b difference, and correlations with RES5/RES6	Theme frequencies; Pearson/Spearman *r*
Order dependence (design validation)	Scenario × sequence interaction in the mixed RM-ANOVA; position-adjusted linear mixed model (autonomy + serial position, random intercepts)	*F*, corrected *df*, *p*, partial η^2^ (90% CI); adjusted vs. unadjusted scenario means
Functional form of the autonomy effect	Orthogonal polynomial contrasts (linear, quadratic, cubic); Holm-corrected adjacent-pair contrasts	*F*, *p*, partial η^2^ per contrast; share of the omnibus effect
Autonomy vs. technical complexity	Mixed RM-ANOVA on MAN2 (sequence between); primary models re-estimated with MAN2 as a time-varying covariate	*F*, corrected *df*, *p*, partial η^2^; adjusted scenario means
Between-person heterogeneity (exploratory)	Random-slope linear mixed model; latent-class growth analysis of individual trajectories	Likelihood-ratio χ^2^, *p*; random-slope SD; class sizes, BIC, entropy

Abbreviations: RM-ANOVA, repeated-measures analysis of variance; RM-ANCOVA, repeated-measures analysis of covariance; *df*, degrees of freedom; GG, Greenhouse–Geisser correction; ε, epsilon; CI, confidence interval; η^2^, eta squared; *dz*, Cohen’s effect size for dependent-samples comparisons; *f*^2^, Cohen’s effect size for the interaction; χ^2^, chi-square statistic; *w*, effect size for the goodness-of-fit chi-square test; HHI, Herfindahl–Hirschman index; B, unstandardized regression coefficient; *SE*, standard error; β, standardized regression coefficient; *R2*, coefficient of determination; VIF, variance inflation factor; SD, standard deviation; BIC, Bayesian information criterion; *r*, correlation coefficient; B1, AI familiarity item; P1–P4b, preference items; RES5 and RES6, responsibility-principle items; MAN1 and MAN2, manipulation-check items.

## Data Availability

Anonymized data, the research instrument, and analysis scripts will be made openly available on the Open Science Framework upon completion of the study. No data have been collected at the time of protocol publication. The Romanian original of the instrument, the statistical analysis plan, and the survey implementation and randomization scripts will be deposited there together with the pre-registration, and the OSF registration link will be added before data collection begins.

## Appendix A. Research Instrument (English Translation of the Romanian Original)

The questionnaire below is the final version of the instrument, translated from the Romanian original that was administered to participants; the Romanian original will be deposited on OSF. Items are shown with their variable codes. Unless otherwise indicated, scaled items use a seven-point end-anchored response format and all closed items are mandatory. The questionnaire is delivered as a single online form; page breaks and branching are indicated in brackets.

### Appendix A.1. Informed Consent and Screening (Page 1)

Study title shown to participants: “Attitudes toward artificial intelligence in facial reconstructive surgery.”

Consent information text: “Hello. We invite you to participate in a scientific research study on attitudes toward the use of artificial intelligence in facial reconstructive surgical planning. Before deciding whether you wish to participate, please read the information below carefully. (1) Nature and objective of the study. This study is academic research examining attitudes, trust, risk perception, and responsibility attribution among adults in Romania toward four progressive levels of artificial intelligence (AI) autonomy in facial reconstructive surgery. (2) What participation involves. Participation involves completing an online questionnaire that records no name, e-mail address, or other detail that could identify you, with an estimated duration of 15–20 min. You will read four short hypothetical scenarios and answer the same set of questions after each. It involves no medical intervention and no physical risk—only questions about opinions, attitudes, and general experiences. (3) Voluntary participation and the right to withdraw. Participation is entirely voluntary. You may decline participation without any consequence and may stop completing the questionnaire at any time, without explanation. If you stop before the end, the answers entered up to that point are not saved and will not be used in the analysis. Because no identifying detail is recorded, after submission your responses can no longer be traced back to you and withdrawn individually. (4) Anonymity and confidentiality. No identifiable data are collected: no name, address, e-mail address, telephone number, or other identifiers. Data are stored on secure servers, in accordance with the GDPR, and will be analyzed exclusively in aggregated, anonymized form. The anonymized data will be made openly and transparently available. (4a) Questions concerning your health. Two questions ask whether you have ever had an operation and, if so, whether it involved the face, head, or neck. These are questions about your health, and under data-protection law they may be processed only with your explicit consent. You give that consent by ticking the second box below. It is optional: you may take part in the study without it, and if you do not tick it, these two questions will not be shown to you. (5) Risks and benefits. The risks associated with participation are minimal. There are no direct benefits to participants. (6) Ethics approval and contact. The study is conducted within the doctoral program of the “Carol Davila” University of Medicine and Pharmacy, Bucharest, and was approved by the UMFCD Research Ethics Committee. Principal investigator: Valeria Gorea Dina, AIFACE-RO project. Questions: aiface.ro@gmail.com.” The consent declaration and the screening items shown on this page are listed in Table A1.

**Table A1:** Consent and screening items.

Code	Item	Response Options
CONS	Consent declaration: “By ticking the option below and continuing to the next page, I confirm that I have read and understood the information above, that I am at least 18 years old, and that I agree to participate voluntarily in this study.”	□ I have read and understood the information above, and I agree to participate. (required)
S1	Do you currently live in Romania?	Yes/No [No → ineligible closing page]
S2	Are you 18 years of age or older?	Yes/No [No → ineligible closing page]
CONS_H	Explicit consent for the health-related questions: “I also agree that my answers to the two questions about previous surgery, including surgery of the face, head, or neck—which are questions concerning my health—may be processed for the purposes of this study (Art. 9(2)(a) GDPR).”	□ I agree. (Optional; if this box is not ticked, questions B4 and B4a are not displayed.)

Ineligible closing page: “Thank you. This study is intended exclusively for residents of Romania aged at least 18 years.” [Form is submitted without further questions.]

### Appendix A.2. Background and Prior Experience (Page 2)

The background and prior-experience items administered on this page are listed in Table A2.

**Table A2:** Background and experience items.

Code	Item	Response Format
B1	How familiar are you with artificial intelligence technology in general?	1 = Not at all familiar… 7 = Extremely familiar
B2	Which of the following descriptions best matches your experience with artificial intelligence tools (e.g., virtual assistants, navigation apps, chatbots)?	I use them regularly (several times a week or more often)/I have used them occasionally (a few times in the past year)/I know they exist, but I have not used them/I know very little about what artificial intelligence tools are
B3	How confident are you that you can understand medical information? (For example: reading a laboratory report, understanding a physician’s explanations, or completing an admission form.)	1 = Not at all confident… 7 = Extremely confident
B4	Have you ever undergone a surgical intervention? [displayed only if CONS_H is ticked]	Yes/No/Prefer not to answer [Yes → B4a; otherwise skip to B5]
B4a	Was it an intervention involving the face, head, or neck?	Yes/No/Prefer not to answer
B5	In the past 12 months, how often have you interacted with the healthcare system (consultations, tests, treatments, hospitalizations)?	Not at all/Once or twice/A few times (3–6 times)/Frequently (monthly or more often)/Prefer not to answer
B6	How much do you trust the Romanian healthcare system to provide you with safe and competent care?	1 = Not at all… 7 = Completely/Prefer not to answer

### Appendix A.3. General Attitudes Toward AI in Medicine (Page 3)

Instruction: “Please indicate the extent to which you agree with each of the following statements.” Response format for G1–G4: 1 = Strongly disagree… 7 = Strongly agree. The four items are listed in Table A3.

**Table A3:** General attitude items.

Code	Item
G1	In general, artificial intelligence will improve the quality of medical care.
G2	I am worried about the involvement of artificial intelligence systems in important medical decisions. (reverse-keyed)
G3	It is important for a physician to remain in control whenever artificial intelligence is used in a surgical intervention.
G4	The use of artificial intelligence in high-risk medical situations may bring dangers that are difficult to foresee. (reverse-keyed)

### Appendix A.4. Attention Check 1 (Page 4)

ATT1 (instructed response, 1–7 scale): “We want to make sure that responses are being recorded correctly. For this question only, please select the answer marked 2, regardless of your opinion. Statement: There should be clear rules on how artificial intelligence is used in medical care.” (1 = Strongly disagree… 7 = Strongly agree; correct response: 2.)

### Appendix A.5. Scenario Blocks (Pages 5–8, Counterbalanced Sequence)

Each scenario page shows the instruction “Please read the following scenario carefully. You will be asked about it later,” followed by the vignette text and the comprehension-check question. A first incorrect comprehension response triggers re-presentation of the same scenario with the note “The selected answer does not match the scenario. Please read it once more, carefully.” The question is then repeated, and the questionnaire continues regardless of the second answer. The response block (Table A4) follows each scenario. The four scenario pages are administered in the sequence to which the participant was randomly allocated—A → B → C → D, B → D → A → C, C → A → D → B, or D → C → B → A (Section 2.1)—and the scenario texts, comprehension checks, and response block are identical in every sequence. The allocated sequence and the serial position of each scenario are stored with the record.

Scenario A (Imaging assistance): “You need facial reconstructive surgery following an injury. Before the operation, an artificial intelligence system analyzes your computed tomography (CT) scan and highlights anatomical structures, such as bone density and tissue layers. Your surgeon independently examines the results provided by the AI, interprets them, and makes all decisions regarding the surgical plan without further input from the AI system.” Comprehension check CC_A: “In this scenario, who made all the final decisions regarding the surgical plan?”—The AI system, based on the CT analysis/The surgeon, independently, after examining the AI analysis (correct)/The AI and the surgeon decided together/It is not clear.

Scenario B (Decision support): “You need facial reconstructive surgery following an injury. Before the operation, an artificial intelligence system analyzes your computed tomography (CT) scan and proposes three possible surgical plans, each with different advantages and disadvantages. Your surgeon examines all three options, chooses the most suitable one, and may adjust it before performing the operation.” Comprehension check CC_B: “In this scenario, who selected the final surgical plan?”—The AI system automatically selected the optimal plan/The patient chose their preferred plan/The surgeon chose among the options proposed by the AI (correct)/It is not clear.

Scenario C (Outcome simulation): “You need facial reconstructive surgery following an injury. The artificial intelligence system analyzes your computed tomography scan and creates 3D simulations showing how your face might look after each surgical option. The surgeon examines these simulations, discusses them with you, and then decides the final surgical plan, taking the simulations into account.” Comprehension check CC_C: “What was the main role of the AI system in this scenario?”—Generating visual simulations of the estimated surgical outcomes (correct)/Automatically selecting the best surgical option/Performing the surgical intervention itself/It is not clear.

Scenario D (Autonomous planning): “You need facial reconstructive surgery following an injury. The artificial intelligence system analyzes your computed tomography scan and generates a complete, optimized surgical plan. Your surgeon examines the plan before the operation. Most of the time, the surgeon operates without modifying the plan; adjustments to the AI proposal are rare.” Comprehension check CC_D: “In this scenario, who generated the surgical plan that was most likely to be followed?”—The surgeon created his or her own independent plan/The AI generated the plan; the surgeon rarely modified it (correct)/The patient and the surgeon created the plan together/It is not clear.

**Table A4:** Per-scenario response block (administered identically after each of the four scenarios; codes carry the scenario suffixes _A, _B, _C, and _D).

Code	Item	Anchors (1… 7)
ACC1	I would accept this form of AI being used in my surgical care.	Strongly disagree… Strongly agree
ACC2	I would feel comfortable with this level of AI involvement in my treatment.	Strongly disagree… Strongly agree
ACC3	I would recommend that a person close to me accept the use of AI in this way, if they needed a similar intervention.	Strongly disagree… Strongly agree
TRU1	I would trust this AI system for planning my surgical intervention.	Strongly disagree… Strongly agree
TRU2	This AI system seems reliable for the task it performs.	Strongly disagree… Strongly agree
TRU3	I would trust that the results provided by this AI system are correct.	Strongly disagree… Strongly agree
RSK1	I consider this level of AI involvement to be safe for patients. (reverse-scored: 8 − score)	Strongly disagree… Strongly agree
RSK2	I am worried about the risk of errors at this level of AI involvement.	Strongly disagree… Strongly agree
RSK3	This way of using AI involves risks that are too great for the patient.	Strongly disagree… Strongly agree
RES1	The surgeon should bear the main responsibility for any error.	No responsibility… Full responsibility
RES2	The hospital or clinic should bear the main responsibility for any error.	No responsibility… Full responsibility
RES3	The company that developed the AI software should bear the main responsibility for any error.	No responsibility… Full responsibility
RES4	A potential error would largely reflect unavoidable medical uncertainty.	No responsibility… Full responsibility
RES5	Even if the surgeon were not legally at fault, he or she still has the moral duty to verify the correctness of the results provided by the AI.	Strongly disagree… Strongly agree
RES6	The responsible parties should be held accountable regardless of the outcome of the surgical intervention, favorable or unfavorable.	Strongly disagree… Strongly agree
MAN1	In the scenario you have just read, how much control did the AI system have over the final surgical decision?	No control… Full control
DIF1	How easy or difficult was it to understand this scenario?	Very easy… Very difficult
MAN2	How technically advanced or complex did the artificial intelligence system in this scenario seem to you?	Not at all advanced… Extremely advanced

Note: RES1–RES4 are introduced by the section header “Attribution of responsibility—If an error occurred in this scenario, how much responsibility should each of the following bear? Responsibility can be shared: please rate each one separately, and do not treat the four ratings as parts of a single total. By responsibility we mean who should answer for the error and put it right—not who would be found at fault in a court of law.” MAN1, DIF1, and MAN2 are introduced by the header “About the scenario you have read.”

### Appendix A.6. Overall Preferences and Acceptability (Page 9)

The overall preference and general acceptability items are listed in Table A5.

**Table A5:** Preference and general acceptability items.

Code	Item	Response Format
P1	Considering all the forms of artificial intelligence involvement you have read about—from imaging assistance to autonomous planning—which configuration would you prefer for your own facial reconstructive surgery?	Configuration 1: AI assists only with imaging—the surgeon makes all decisions/Configuration 2: AI proposes surgical options—the surgeon chooses/Configuration 3: AI generates outcome simulations—the surgeon decides after examining them/Configuration 4: AI generates the surgical plan—the surgeon examines it but rarely modifies it/I would prefer AI not to be involved in any way/I have no preference/I cannot decide
P2	If you have any concerns or reservations about the involvement of artificial intelligence in facial surgery, please describe them briefly here. (Optional)	Free text, maximum 500 characters
P3	Overall, how acceptable is the use of artificial intelligence in facial reconstructive surgery, regardless of the degree of involvement?	1 = Completely unacceptable… 7 = Completely acceptable
P4a	The surgeon should always remain legally responsible for any surgical outcome, regardless of the degree of artificial intelligence involvement.	1 = Strongly disagree… 7 = Strongly agree
P4b	The surgeon should always remain ethically responsible for any surgical outcome, regardless of the degree of artificial intelligence involvement. [Presented on the same page and scale as P4a, immediately below it.]	1 = Strongly disagree… 7 = Strongly agree

### Appendix A.7. Attention Check 2 (Page 10)

ATT2: “This question checks whether you have read the scenarios carefully. What type of surgical intervention was described in the scenarios you read?”—Cardiac bypass surgery/Knee replacement/Facial reconstructive surgery (correct)/Laser eye correction.

### Appendix A.8. Demographics (Page 11)

The demographic items are listed in Table A6.

**Table A6:** Demographic items.

Code	Item	Response Format
D1	What gender do you identify with?	Male/Female/Non-binary or another gender identity/Prefer not to answer
D2	What is your age (in completed years)?	Numeric entry, 18–99
D3	What is the highest level of education you have completed?	High school or below/Vocational school or technical qualification/Bachelor’s degree or equivalent/Master’s degree/Doctorate or medical degree (PhD, MD, etc.)
D4	Which of the following best describes your current or most recent field of activity?	Healthcare or medicine/Education or academia/Business, management, or administration/Legal services or liberal professions/Industry, engineering, or manufacturing/Other
D5	Compared with the average person in Romania, how would you describe your current financial situation?	1 = Well below average… 7 = Well above average
D6	In what type of area do you currently live?	Urban/Rural/Prefer not to answer
D7	In which region of Romania do you currently live?	Bucharest–Ilfov/North-East/South-East/South–Muntenia/South-West Oltenia/West/North-West/Center/Prefer not to answer

### Appendix A.9. Debriefing (Closing Page)

“Thank you for participating! This study examines how adults in Romania perceive four different levels of artificial intelligence involvement in facial reconstructive surgery. You read and evaluated all four scenarios, presented in one of four possible orders, to which you were assigned at random. The scenarios are hypothetical: in current clinical practice in Romania, final surgical decisions belong to the physician, and highly autonomous AI systems of the kind described in some scenarios are not in use. Your responses carry no detail that could identify you and will be analyzed exclusively in aggregated form. For questions or to request results after publication, please contact: aiface.ro@gmail.com.”
